# The Epigenetic Link between Polyphenols, Aging and Age-Related Diseases

**DOI:** 10.3390/genes11091094

**Published:** 2020-09-18

**Authors:** Itika Arora, Manvi Sharma, Liou Y. Sun, Trygve O. Tollefsbol

**Affiliations:** 1 Department of Biology, University of Alabama at Birmingham, Birmingham, AL 35294, USA; itiarora@uab.edu (I.A.); manvi@uab.edu (M.S.); sunlab@uab.edu (L.Y.S.); 2 Comprehensive Center for Healthy Aging, University of Alabama Birmingham, 1530 3rd Avenue South, Birmingham, AL 35294, USA; 3 Comprehensive Cancer Center, University of Alabama Birmingham, 1802 6th Avenue South, Birmingham, AL 35294, USA; 4 Nutrition Obesity Research Center, University of Alabama Birmingham, 1675 University Boulevard, Birmingham, AL 35294, USA; 5 Comprehensive Diabetes Center, University of Alabama Birmingham, Birmingham, AL 35294, USA

**Keywords:** aging, DNA methylation, histone modifications, non-coding RNAs and polyphenols

## Abstract

Aging is a complex process mainly categorized by a decline in tissue, cells and organ function and an increased risk of mortality. Recent studies have provided evidence that suggests a strong association between epigenetic mechanisms throughout an organism’s lifespan and age-related disease progression. Epigenetics is considered an evolving field and regulates the genetic code at several levels. Among these are DNA changes, which include modifications to DNA methylation state, histone changes, which include modifications of methylation, acetylation, ubiquitination and phosphorylation of histones, and non-coding RNA changes. As a result, these epigenetic modifications are vital targets for potential therapeutic interventions against age-related deterioration and disease progression. Dietary polyphenols play a key role in modulating these modifications thereby delaying aging and extending longevity. In this review, we summarize recent advancements linking epigenetics, polyphenols and aging as well as critical findings related to the various dietary polyphenols in different fruits and vegetables. In addition, we cover studies that relate polyphenols and their epigenetic effects to various aging-related diseases such as cardiovascular diseases, neurodegenerative diseases, autoimmune disorders, diabetes, osteoporosis and cancer.

## 1. Introduction

Aging is an intricate biological process that causes changes in the normal function of an organism throughout its lifetime [1,2,3]. Both genetic and non-genetic factors, which include environmental factors, are implicated in aging by causing structural and molecular modifications at cellular, tissue, and organ levels [4,5]. Numerous animal models have served as a baseline to identify mutations in key genes and the pathways which thereby contribute to aging. For instance, studies have identified that genetic factors such as mutations in mitochondrial DNA cause a decline in somatic stem cell function and lead to mammalian aging [6]. A role of mitochondrial aging was reported in *Caenorhabditis elegans* (free-living transparent nematode living in temperate soil environment and about 1 mm in length) wherein decreased activity of the electron transport chain and adenosine 5′-triphosphate (ATP) synthase resulted into both reduction in body size and increased lifespan [7]. Another biochemical process, oxidative stress/damage (OS) plays a critical role in mammalian aging. Experiments on mouse (*Mus musculus*) found advantageous effects of dietary-based caloric restriction (CR) on brain function and lifespan based on its ability to reduce OS levels [8]. OS arises due to various environmental factors such as metabolic processing of consumed food products with high caloric intake [9] and natural toxic chemicals found in plants [10]. During the process of aging, reactive oxygen species (ROS) produced inside the biotic system induces changes in cellular activities such cell survival thereby inducing OS and inflammatory responses [11,12]. Therefore, increased ROS production and proper functioning of antioxidant defense mechanisms protecting the cells from oxidative damage are endogenous mechanisms in aging-related diseases [13]. Other non-genetic elements such as an unhealthy diet, smoking, alcohol or drug abuse and exposure to noxious chemicals also contribute to ROS mediated aging [14]. In the past, studies have emphasized on numerous health benefits related to the consumption of bioactive compounds on an organism’s health in extending the lifespan and delaying aging.

For instance, studies have examined the effect of dietary polyphenols such as green tea polyphenols (GTPs) on damage to neuronal biomolecules associated with OS and ROS, which are generated during metabolism in neurodegenerative diseases such as Alzheimer’s disease (AD) and Parkinson’s disease (PD) [15]. Polyphenols are secondary metabolites of plants found in fruits, vegetables, and certain beverages which possess strong antioxidant properties and protect against various pathogens [16]. Polyphenols-associated studies are primarily based on their ability to affect critical regulatory molecules involved in various diseases such as cancer [17]. Specifically, dietary polyphenols possess an ability to modulate biological states associated with OS and chronic inflammation that may be primary contributors to different types of cancers including cervical, ovarian and breast cancer. Various studies have also demonstrated the dynamic role of polyphenols in the suppression of inflammation related to cancer development [18]. For instance, a study in *Mus musculus* showed the beneficiary effects of GTPs such as epigallocatechin gallate (EGCG) on ultraviolet based (UVB) induced skin tumors. Additionally, the administration of EGCG in wild type mice led to a decline in levels of inflammation markers such as cyclooxygenase-2 (COX-2) and prostaglandin-E2 associated with tumor development [19].

**Figure 1:**
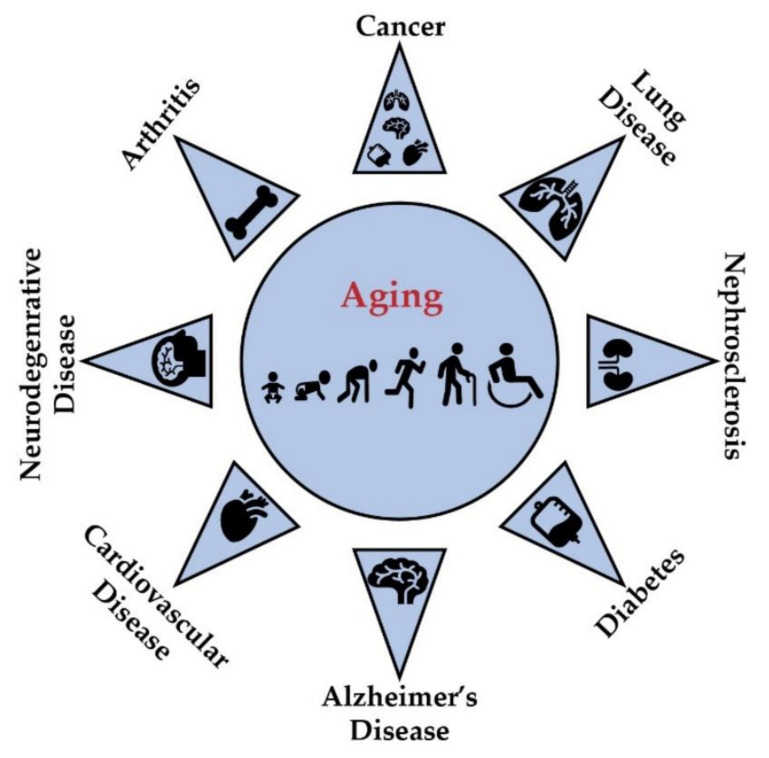
Typical aging and aging-related diseases such as cancer, lung disease, nephrosclerosis, cardiovascular disease, AD, diabetes and arthritis.

In addition, other nutrients and bioactive polyphenols such as resveratrol (RES), curcumin (CUR) and quercetin also have a substantial impact on the aging process in different organisms [20]. Figure 1 illustrates numerous aging-related diseases such as cancer [21], neurodegenerative diseases [22], nephrosclerosis, arthritis and cardiovascular diseases [23] caused by a number of genetic and non-genetic factors.

Although aging and age-related diseases are associated with profound changes in epigenetic patterns, less is known about the role of dietary polyphenols in extending lifespan through alterations in epigenetics machinary. Therefore, understanding the epigenetic link between polyphenols and aging is a growing area of research and will provide deeper insight into the development of novel approaches influencing longevity and aging. Hence, this review will focus on understanding the influence of dietary polyphenols on epigenetic modulations and how these changes in-turn impact aging and longevity. Additionally, this review will also provide aggregate information on various studies related to influence of dietary polyphenols and epigenetic modifications in age-related diseases. In this review, the term epigenetics will be used broadly, highlighting the genomic alterations contributing to DNA methylation patterns, histone modifications and non-coding RNAs (ncRNAs).

## 2. Dietary Polyphenols

Depending on their number of classes of phenolic rings, polyphenols are classified into distinctive groups which comprise structural elements that allow these rings to bind to each another. Phenolic acids, flavonoids, stilbenoids and lignans are principal classes of polyphenols [24]. Phenolic acids are found in vegetables, fruits, cereals, olives, legumes and beverages such as coffee and tea. Phenolic acids are classified into two major classes based on benzoic acid byproducts (such as gallic acid and egallic acid) and cinnamic acid byproducts (such as caffeic acid and ferulic acid). Due to their wide availability in different sources, phenolic acid exhibits various antioxidant [25], anti-inflammatory [26] and anti-cancerous properties [27].

Flavonoids, another classes of polyphenols, are comprised of 2 aromatic rings that are bound together by 3 carbon atoms and are sub-cateogarized into flavonols, flavones, flavanones, isoflavones, anthocyanidins and flavanols [28]. Flavanones are widely distributed in citrus fruits (such as orange and lemon) and possess positive cardioactive properties [29]. Flavonols are another class of flavonoids that are widely distributed in different fruits and vegetables such as onions, tea, grapes skin, apple, blueberries and wine [30]. Unlike flavonols, flavones are less abundant and mainly found in celery [31] and parsley [32]. Additionally, flavanones are mainly found in high concentrations in tomatoes [33] and citrus fruits [34]. Overall, flavonoids exhibit various antioxidant [35], anti-inflammatory [36] and anti-cancerous properties [37]. For instance, myricetin, a plant based flavonoid beholds antioxidant, anti-inflammatory and anti-cancerous properties against numerous diseases such as cardiovascular diseases and cancer [38]. A study reported that the anti-oxidative property of myricetin reacted 28-times faster with oxygen-centered galvinoxyl radicals in comparison to d-α-tocopherol (ATF), a lipid-soluble antioxidant found in biological membranes. However, the compound was incapable of preventing vitamin E-deficient microsomes from lipid peroxidation [39]. Investigations have also reported that administration of myricetin in combination with other antioxidants such as vitamins C or E in murine melanoma B16F10 cells can induce increased catalase (CAT) activity and decrease superoxide dismutase (SOD) and glutathione peroxidase (GPx) activities [40].

Stilbenoids are another class of polyphenols which are often found in various plant families such as dipterocarpaceae, gnetaceae and fabaceae [41]. RES, pterostilbene (PTER), piceatannol and gnetol are major stilbenoids which exhibit various protective properties against cardiovascular diseases, cancer, atherosclerosis and diabetes by [42]. Besides stilbenoids, lignans are another class of polyphenols, mainly found in tea, coffee and olive oil that also possess anti-cancerous, and antioxidant properties [43,44,45]. Table 1 summarizes various classifications of polyphenols, their major sources, their relative content within these sources, and proposed molecular functions [46].

## 3. The Link between Epigenetics and Aging

The term epigenetics is associated with specific gene expression patterns observed during heritable changes due to mitotic and meiotic activities which affect multiple processes such as cell development, cell differentiation and X-chromosome inactivation. The key reason for understanding and defining epigenetics is to illuminate specific epigenetics changes that occur during aging and to also comprehend whether the changes depend on genetic, environmental or stochastic factors [83]. Epigenetic modifications are comprised of DNA methylation, histone post-transcriptional modifications such as methylation, acetylation, ubiquitination and phosphorylation and ncRNAs. These alterations play a vital role in the healthy development of an organism as they are crucial for various biological processes such as transcription, cell division, DNA replication and many others. Therefore, the overall stability of epigenetic mechanisms is crucial for maintenance of proper molecular activity, which reduces the possibility of various diseases and further delays the aging process [84].

### 3.1. DNA Methylation and Aging

Methylation at cytosine residues serves as a pivotal epigenetic factor that modulates gene expression in various organisms. These changes occurs as a result of de novo DNA methyltransferases (DNMTs) enzymes activity which are primarily responsible for transferring methyl group to 5 carbon position of cytosine [85]. Numerous experiments have revealed that there is a significant decrease in global 5-methylcytosine (5mC) during ontogenesis. For instance, in 1967, it was discovered that an overall reduction of 5mC level occurs during ontogenesis at different stages in humpback salmon [86]. One study in humans (*Homo sapiens*) identified aging-associated DNA methylation changes at CpG sites (DNA regions wherein cytosine nucleotide is followed by guanine nucleotide in a linear sequence of bases in the 5′ → 3′ direction) in different tissues such as blood, brain and kidney. The study reported that tissue-specific age-associated CpGs (ageCGs) decreased methylation and are localized outside CpG islands. Unlike, tissue-specific ageCGs, common ageCGs induces increased methylation and are primarily situated inside the CpG islands [87].

Currently, DNA methylation during aging occurs due to two contradicting phenomena consisting of epigenetic drift and the epigenetic clock. The epigenetic clock is built based on supervised machine learning methods which aim to identify epigenetic methylation changes that are closely related to chronological age. The supervised machine learning approach helps to determine informative CpGs for age prediction thereby associating DNA methylation (DNAm) levels to age estimates [88,89]. The age estimators, also known as “clock CpGs”, are associated with a mathematical algorithm based on a Pearson correlation coefficient (r) referring to “age correlation” and median error that calculates the absolute difference between the chronological age and biological age. These coefficients surpass a confidence interval (CI) of 95% within multiple tissues for individuals with their ages within 0-100 years [90]. The age estimator evaluates the DNAm age (also known as epigenetic age) within cells and tissues. Epigenetic age is also known as DNAm age which emphasizes crucial facets of genetic age and further reveals a strong correlation with age-related conditions, thereby predicting an individual’s age. This phenomenon relates to a wide range of aging-related changes which contribute to various diseases such as cancer [91]. In the past, studies have provided strong evidence suggesting the importance of polyphenols-associated changes in DNA methylation as enumerative measures of long-term dietary exposure to certain stilbenoids such as RES and pterostilbene (PTER) in nutritional epidemiology and clinical experiments. The study demonstrated that treatment of MCF10A human mammary epithelial cells with RES and PTER at 15 µM for 9 days led to subtle alterations thereby suggesting remodelling of DNA methylaton patterns at eight CpG sites located within *KCNJ4, RNF169*, *BCHE*, *DAOA*, *HOXA*, *RUNX3*, *KRTAP2-1* and *TAGAP* [92].

Epigenetic drift refers to progressive deviation in DNA methylation levels during aging, which begins immediately after birth and becomes more prevalent during pre-puberty. Epigenetic drift can also be affected by various genetic factors and environmental factors, thereby evolving as a potential biomarker for different biological factors affecting aging and leading to disease progression. Therefore, it is quite crucial to understand the mechanism for these fluctuations [93]. These findings indicate that alterations in DNA methylation could potentially influence various transcription factors at specific sites, eventually contributing to the dysregulation of gene expression during aging. Table 2 lists different proteins that contribute to DNA methylation and their function at the molecular and biological levels.

### 3.2. Histone Modifications and Aging

The histone machinery of chromatin is linked to a wide variety of translational and post-translational modifications. Histone modifications are primarily comprised of acetylation, methylation, phosphorylation and adenonsine diphosphate (ADP) ribosylation. Histone modifications are known to either interrupt the chromatin organization or offer new binding surfaces for the deployment of various proteins in a specific region of chromatin [99]. Histone acetyltransferases (HATs), histone deacetylases (HDACs) and histone methyltransferases (HMTs) are key enzymes that influence transcriptional machinery by chemical modifications of histones. For example, a study in *Saccharomyces cerevisiae* (baker’s yeast) demonstrated that decrease in Silencing Information Regulator 2 (Sir2) protein, which is responsible for maintaining silencing in yeast hetrochromatin telomeric regions, and increased levels of histone 4 on lysine 16 acetylation (H4K16Ac) delayed aging and extended the lifespan [100]. Another study in *S. cerevisiae* reported that deletion of the histone acetylase *RPD3* gene extended the lifespan by enhancing silencing at three hetrochromatin regions of the genome; silent mating type (HM), subtelomeric region and ribosomal DNA (rDNA) [101].

Besides HDACs, HMTs also play a critical role in regulating transcriptional activities. An investigation in *S. cerevisiae* demonstrated a role for histone 3 lysine 36 (H3K36) methylation in promoting longevity by increasing transcription fidelity. The study demonstrated that the deletion of the K36me2/3 demethylase *Rph1* gene led to increasing levels of H3K36me3 further extending the lifespan [102]. Additionally, studies in *C. elegans* have also suggested that the inactivation of somatic Set-26 domain caused a robust extension of lifespan and changes in the levels of histone 3 on lysine 9 trimethylation (H3K9me3) and histone 3 on lysine 27 trimethylation (H3K27me3) thereby inducing chromatin structure restoration and promotion of longevity [103]. Therefore, based on these results, the process of histone modification is complex and can have a positive or negative impact on transcriptional machinery, eventually extending the lifespan. Table 3 demonstrates histone modification activities of HATs, HDACs and HMTs enzymes with their exact molecular events and biological function.

### 3.3. Non-Coding RNAs and Aging

Besides DNA methylation and histone modifications, ncRNAs also play a vital role in maintaining genomic stability by regulating gene expression. Primary research on various organisms, such as *H. sapiens*, explains that ncRNAs-related regulatory processes contribute to the pathogenesis of various aging-related diseases. NcRNAs regulates a wide range of key cellular processes such as translation and gene expression profiling. Among different types of ncRNAs, microRNAs (miRNAs) and long non-coding RNAs (lncRNAs) play a central role in maintaining the healthy lifespan of organisms. Table 4 provides a list of different ncRNAs and their aging-associated molecular activity and related biological functions.

One of the critical factors during the aging process is genomic instability which can be stimulated in part by changes in miRNAs, and many have reported the role of various miRNAs in modulating lifespan and regulating tissue aging. The best-characterized examples of miRNAs functions during aging are derived from *C. elegans* investigations. A study conducted in *C. elegans* emphasized the modulating effects of miRNA lin-4 and lin-14 thus regulating the insulin/insulin-like growth factor-1 pathway. As a result, reducing of activity of lin-4 reduced the lifespan and improved tissue aging. Furthermore, overepxression of lin-4 and reduced expression of lin-14 extended the life span [114]. Besides miRNAs, molecular mechanisms of lncRNAs associated with transcriptional, translational and post-translational activity can affect aging-related pathways [115]. A study in *Drosophila melanogaster* (common fruit fly) demonstrated lncRNAs-mediated regulation of aging-associated pathways at 7 days and 42 days during CR and well-fed conditions thereby prolonging the lifespan. As a result, 1406 differentially expressed coding genes and 102 differentially expressed lncRNAs were identified. A recent aging study using fruit flies revealed that both short-term and long-term CR diets had intriguing impact on novel lncRNAs expression in addition to their well-studied transcriptional effects. Consequently, Gene ontology (GO) and KEGG (Kyoto encyclopedia of genes and genomes) analysis identified novel aging-associated pathways during CR such as protein processing pathways, hippo signaling pathway-fly and phototransduction-fly. Furthermore, novel lncRNAs XLOC_092363 and XLOC_166557 positioned in a 10 kb upstream regions of the *hairy* and *ems* promoters were also identified. The study also reported that lncRNA XLOC_076307 silencing induced growth arrest and DNA damage inducible alpha (Gadd45A) associated expression levels changes [116]. Altogether, these studies suggest that ncRNAs could serve as a useful resource for understanding aging and aging-related diseases.

## 4. Effects of Dietary Polyphenols the Epigenetic Machinery and Aging

Even though OS and CR play a pivotal role in aging, it is also imperative to understand the underlying mechanism by which different bioactive compounds imitate the effects of CR and further reduce the risks associated with CR side effects. A plethora of studies have demonstrated the positive effects of polyphenolic compounds such as apigenin, CUR, EGCG, genistein (GES), PTER and RES on aging-related phenomena by facilitating anti-cancer and anti-inflammatory effects as discussed below. The influence of an environmental factor such as exercise and diet on gene expression and longevity in different organisms has been a topic of great interest [117]. Diet plays a key role in regulating epigenetic modifications such as DNA methylation, DNA demethylation and histone modifications regulated through HDACs, HATs and HMTs enzymes [118,119]. For instance, a study demonstrated the effects of RA and sodium butyrate (BUT) on gene expression and HDAC activity in human RA-resistant A375 and RA-responsive S91 murine melanoma cell lines. As a result, BUT treatment resulted in both *RARβ* and *p21^waf1/cip1^* mRNA being expressed in A375 cells, and only *p21^waf1/cip1^* mRNA expression being expressed in S91 cells. Moreover, combined administration of RA and BUT caused synergistic activation of RA-responsive reporter gene in S91 cells but not in RA-resistant A375 cells. Treatment with BUT increased histone H4-acetylation levels in both RA-resistant A375 and RA-responsive S91 cell lines [120]. Another study revealed the effect of diallyl disulfide (DADS), an organosulfur compound primarily found in garlic, on HDAC activity and gene expression changes in human colon tumor Caco-2 and HT-29 cell lines. Treatment with DADS led to increasing histone H3 acetylation in both Caco-2 and HT-29 cell lines and histone H4 hyperacetylation at lysine 12 and lysine 26 in Caco-2 cell lines. Furthermore, treatment with DADS also enhanced the expression of *p21^waf1/cip1^* at mRNA and protein levels in both Caco-2 and HT-29 cell lines [121]. Either of these studies provides strong evidence implicating the role of dietary polyphenols and their impact on aging-associated histone modifications. Therefore, it is vital to understand histone modifications as a molecular target mechanism linking diet related changes and longevity.

### 4.1. Apigenin

Apigenin is a plant-based flavone with anti-oxidative properties. Studies have demonstrated the protective effects of apigenin on aging-related diseases such as colon cancer, skin cancer and many others. A study investigated protective effects of apigenin where mice were administered continuously for 9 weeks with D-galactose subcutaneously. As a result, dietary treatment with apigenin resulted in improved aging-related changes such as behavioral deterioration, decreased organic index, histopathological injury, increased senescence-associated activity of β-galactosidase (SAβ-gal), enriched glycation product (AGE) level and decreased levels of MDA. Furthermore, treatment with apigenin also caused up-regulation of HO-1 and NQO-1, downstream gene targets for the Nrf2 pathway, eventually delaying the process of aging [122]. Another study on skin showed that apigenin reinstated the viability of human dermal fibroblasts (nHDFs) which decreased after exposure to ultraviolet (UV) radiation. Additionally, apigenin treatment also reduced collagenase and matrix metalloproteinase (MMP)-1 expression in nHDFs irradiated by UVA [123]. The study also demonstrated the beneficial effects of apigenin on texture of the skin, moisture and the depletion of transepidermal water (TEWL). As a consequence, apigenin-containing cream increased skin evenness, moisture content and TEWL. In addition, consistent use of apigenin-containing cream eventually decreased wrinkle length and further boosted dermal density and dermal elasticity. In another study, HT-29 and HCT-15 cell lines of colorectal cancer were treated with apigenin, which resulted in anti-proliferative and apoptotic effects by inducing biochemical and morphological changes. Treatment with apigenin led to increasing production of free radical species, inhibition of retinoblastoma phosphorylation and up-regulation of *p21*, eventually suppressing cyclin D1 and E activity [124]. These changes attributed to dietary treatment with apigenin suggest that it is a potent dietary phytochemical in chemoprevention and aging-related studies.

### 4.2. Curcumin (CUR)

CUR is another potent polyphenolic compound that is mainly found in vegetables and primarily known for its anti-oxidative properties influencing chronological and replicative aging. In the past, studies have explained the anti-aging properties of CUR in the context of adipose tissue-derived mesenchymal stem cells (rADSCs) by *TERT* gene expression in rats. rADSCs were isolated from adipose tissues and treated with CUR in a dose-dependent manner (1 µM–20 µM). As a result, dietary treatment with CUR resulted in considerable proliferation of rADSCs after 48 h of treatment at 1 µM and 5 µM CUR concentrations, thereby lessening population multiplying time and rADSCs aging at different cell passages. Moreover, SA-β-gal staining results indicated that CUR significantly decreased the number of senescent cells in different passages (five and seven cell passages) and also increased the expression of *TERT* gene at 1 µM and 5 µM CUR concentrations [125]. Additionally, a study in rats (*Rattus rattus*) investigated the beneficial effects of CUR on muscle force characterstics in aging rats. 32 weeks old F344xBN rats were administered with purified diet of CUR (AIN-93M) for 4 months. First group was administered with control ad libitum diet (CON, *n* = 5), second group was administered with purified CUR diet (CUR, *n* = 4) and third group, pair fed (PAIR, *n* = 4), received a purified diet in the quantity equal to group 2 (CUR). Body mass, muscle mass and contractive characteristics were evaluated using independent sample t-tests. As a result, PAIR group exhibited lower muscle mass unlike CUR group which had identical muscle mass. The study also reported that peak tetanic tension was higher in CUR group in comparison to PAIR group.

In addition, CUR also posses anti-inflammatory, anti-proliferative and anti-cancerous properties [126,127]. For instance, CUR inhibited DNMT1 activity in human breast cancer MCF-7 cell lines. Additionally, treatment with CUR also led to reactivation of Ras-associated domain family protein 1A (RASSF1A) and further decreased cell proliferation and tumor growth [128]. In addition, a study in human lung cancer A549 cell lines reported that treatment with CUR increased RA receptor beta (*RARβ*) gene expression, decreased tumor growth and DNMT3b activity [129]. To better understand the association of dietary polyphenols and DNA methylation, it is critical to gain a better understanding of underlying mechanisms between DNA methylation, gene expression and various aging-related diseases.

### 4.3. Epigallocatechin-3-Gallate (EGCG)

EGCG is the primary bioactive compound found in green tea. EGCG is widely studied for its demethylating properties through its action as a DNMT inhibitor in various lung cancer, leukemia, breast cancer as well as other neurodegenerative disorders. A study was conducted in human lung adenocarcinoma A549 cell lines wherein an A549/DDP cell line model was designed by treating A549 cells with a higher concentration of cisplatin (DDP). The investigation determined that the administration of EGCG in A549/DDP cell lines resulted in inhibition of cell proliferation, cell cycle arrest in the G1 phase and increased apoptotic activity. Furthermore, EGCG treatment led to inhibition of DNMT and HDAC activity and down-regulation of Growth arrest specific 1 (*GAS1*), TIMP metallopeptidase inhibitor 4 (*TIMP4*) and Intracellular adhesion molecule 1 (*ICAM1*) genes [130]. Another study indicated that the administration of EGCG in human breast cancer MCF-7 and leukemia HL90 cell lines resulted in decreased cell proliferation and also induced apoptosis in both the cell lines. Additionally, EGCG treatment in MCF-7 cells caused a decrease in *hTERT* promoter methylation and inhibition of histone 3 lysine 9 (H3K9) acetylation. As a result, EGCG was identified as a pivotal polyphenol possessing anti-oxidative properties, altering epigenetic mechanisms and eventually causing cell death in both MCF-7 and HL60 cell lines [131]. Subsequently, another investigation demonstrated that administration of EGCG in adult hippocampal neural progenitor cell (NPC) cultures and in denate gyrus of adult mice improved spatial cognition thereby promoting adult neurogenesis. EGCG treatment caused a significant increase in the total number of 5-Bromo-2′-deoxyuridine (BrdU)-labeled cells and increased expression of the Ssh mRNA receptor as well as *Gli1*, a downstream transcriptional target for Ssh [132]. Another report indicated that EGCG extracted from green tea decreased 5mC, mRNA and protein levels of DNMT1, DNMT3a and DNMT3b in human epidermoid carcinoma A431 cells. Furthermore, treatment with EGCG also decreased histone deacetylase activity and increased acetylation of lysine 9 and 14 on histone H3 [133]. Another study also suggested that EGCG inhibits DNMT activity and reactivates methylation-silenced genes in HT-29 cell lines of human colon cancer, KYSE-150 cell lines of esophageal cancer and PC3 cell lines of prostate cancer [134]. These results together suggest that the beneficial properties of EGCG are numerous with respect to aging.

### 4.4. Genistein (GES)

Besides apigenin and CUR, GES is another polyphenol that is associated with aging. GES is mainly found in olives, soybeans, fava beans and is known for its wound healing properties and photoprotective properties. GES also possess inhibitory properties for tyrosine kinases by controlling glycosaminoglycan (GAG) synthesis and increase stability against UV-light exposure. A study in UVB-irradiated human skin fibroblast BJ-5ta cells suggested that combinatorial administration of GES with daidzein led to a decrease expression of COX-2 and induced growth arrest, thereby exerting a photoprotective effect on UVB-irradiated human skin fibroblast BJ-5ta cells [135]. GES was also tested for protection against UV-light exposure in human skin HaCat cells wherein administration of GES led to the decreased inflammatory activity by suppressing the basal and stimulated COX-2 mRNA and protein expression levels [136]. Subsequently, a report in *Mus musculus* demonstrated the anti-renal fibrosis activities of GES by restoring *klotho* promoter activity, a kidney-enriched anti-aging and fibrosis-suppressing protein. As a result, administration of GES led to restoration of *klotho* promoters by inhibiting histone 3 deacetylation as well as inhibiting DNMT1 and DNMT3a activity [137]. A study also demonstrated anti-senescence activity of GES in human umbilical vein endothelial cells (HUVECs). HUVECs were treated with GES at 1000 nM concentration for 30 min and exposed to 50 mg/L of ox-LDL for 12 h. As a result, treatment with GES inhibited ox-LDL-induced cellular senescence by decreasing p16, p21 protein levels and further decreasing SA-β-gal activity. Additionally, the study revealed the association of SIRT1/LKB1/AMPK pathway with GES in enhancing autophagic flux by increasing LC3-II levels and decreasing p62, p-mTOR and p-P70S6K levels [138]. Therefore, administration of GES in a dose-dependent manner could be helpful in establishing specific concentrations for clinical efficacy and further delaying aging.

### 4.5. Pterostilbene (PTER)

PTER, a natural *trans-*3,5-dimethyl ether, is another class of polyphenolic compound that is frequently found in grapes, berries, peanuts and wine. PTER is known for its towering pharmacokinetics and pharmacodynamics properties. Multiple studies have indicated that the administration of PTER induces chemopreventive effects by targeting metastatsis-associated protein 1 (MTA1) in human prostate cancer. MTA1 is deemed to be a critical upstream regulator of tumorigenesis and targets for *c-Myc* and *Akt,* which are key genes in prostate cancer progression. The study reported that administration of PTER led to inhibition of MTA1, resulting in decreased cell proliferation as well as increased apoptosis and angiogenesis [139]. An additional report explained the antioxidant and myocardial protection property of PTER in the C57BL/6 mice model and H9c2 cell lines. C57BL/6 mice were administered with 20 mg/kg of doxorubicin (DOX), and H9c2 cells were treated with 1 μM DOX. As a result, dietary treatment with PTER led to the reduction of OS thereby improving AMP-activated protein kinase (AMPK) and SIRT1 signaling pathways. The administration of PTER inhibited OS induced by DOX and mitochondrial morphological disorder by up-regulation of peroxisome proliferator-activated receptor-gamma co-activator 1alpha (PGC-1α), thereby activating AMPK and SIRT1 activity by enhancing SIRT1 [140]. Another study examined the combinatorial impact of RES and PTER by altering DNA damage response and their effect on SIRT1 and DNMT expression activity. The results demonstrated that a synergistic combination of RES and PTER induced inhibition of triple-negative breast cancer (TNBC) by reducing cell proliferation, increasing apoptosis, and upregulating cell cycle arrest in HCC1806 and MDA-MB-157 breast cancer cells. Furthermore, combinatorial treatment of RES and PTER also resulted in the down-regulation of DNMT enzyme expression in HCC1806 cells with no effect on DNMT enzyme expression in breast MCF10A control cell lines [141]. These studies suggest that the administration of PTER, along with other polyphenols such as RES, may serve as a therapeutic target for novel treatments of various diseases such as breast cancer.

### 4.6. Resveratrol (RES)

RES, a polyphenol found in almonds, blueberries, and grapes, also possesses potential anti-aging and antidiabetogenic properties. Researchers have studied the metabolic effects of RES results due to inhibition of cAMP-degrading phosphodiesterases eventually causing increased cAMP levels in *Mus musculus.* As a result, treatment with RES induced Epac1 activation, which increased intracellular Ca^2+^ levels and further triggered the CamKKβ-AMPK pathway by controlling phospholipase C and ryanodine receptor Ca^2+^ release channel. Additionally, treatment with RES also increased NAD^+^ and Sirt1 activity, thus, preventing diet-induced obesity and enhancing mitochondrial function, physical stamina, and glucose tolerance in mice [142]. An investigation conducted on *Mus musculus* reported that a low dosage of RES imitated CR effects and also hindered aging parameters in mice. A control diet and a lower dose of RES [4.9 mg kg^−1^day^−1^] and CR diet was administered to mice aged 14 months (middle aged) and 30 months (old age). As a result, RES dietary treatment simulated CR properties by inhibiting gene expression in heart, skeletal muscle and brain tissues, eventually preventing cardiac dysfunction [143]. Another study assessed the effect of RES on physiological changes in middle aged, high-calorie diet mice in comparison to those on a standard diet. Dietary administration of RES extended the lifespan by increasing AMPK and PGC-1α activity, increasing mitochondrial number and further decreasing insulin-like growth factor-1 (IGF-I) levels [144]. These results suggest that RES possesses beneficial properties in treating obesity-related disorders as well as aging-related diseases.

### 4.7. Quercetin

Quercetin is another flavonoid mainly found in vegetables, fruits and actively responsible for maintaining cellular ROS levels. A study in a *Podospora anserine* (a filamentous ascomycete fungus) model demonstrated the role of *S*-adenosylmethionine-dependent *O*-methyltransferase PaMTH1 wherein the treatment with quercetin induced longevity. Administration of quercetin extended the lifespan in wild type *P. anserine* but not in a mutant with PaMTH1 deletion. Additionally, treatment with quercetin also increased mitochondrial respiration and respiratory complexes along with increased release of superoxide anion [145]. Another investigation reported the effect of quercetin in declining the oocyte quality during post-ovulatory aging of mouse oocytes by modulating SIRT expression and MPF activity. As a result, treatment with quercetin repressed aging-associated changes in organization of spindles and distribution of mitochondria. In addition, quercetin treatment prevented overall decrease in SIRT expression and maturation-promoting factor (MPF) activity, and further delayed apoptosis onset during post-ovulatory aging. Moreover, treatment with quercetin during post-ovulatory aging also enhanced the early development of the embryo [146]. These studies suggest that quercetin plays a pivotal role in extending the lifespan in different organisms.

These polyphenols and many others provide evidence that polyphenol-rich diets may provide benefits to an organism’s health and display strong anti-aging properties as well as attenuate the effects of various diseases. Table 5 summarizes the epigenetic and aging-related activity, aging patterns, and their target molecular activity of dietary polyphenols.

## 5. Conclusions

Epigenetic alterations such as DNA methylation, histone modifications, and ncRNAs play significant roles not only as hallmarks of aging-related diseases, but also as important molecular processes that underlie the basis of aging. The main cellular consequences of epigenetic dysregulation are variations in transcriptional activation, transcriptional repression, changes in genomic stability and specific disease-associated morphological changes. A significant challenge in the field of epigenetics and aging-associated diseases is to identify the exact molecular mechanisms that are expected to result in these diseases. Therefore, it is necessary to understand the precise causes behind these changes.

High-throughput studies along with various transcriptomics analysis, have also provided a wealth of information about epigenetic modifications. Furthermore, understanding the significance of dietary polyphenols to an organism’s health provides a deeper understanding of diverse molecular activities related to aging. Evidence suggests that various dietary polyphenols may a lead to an increasing cell proliferation, a decrease in tumor incidence, an increase in tumor latency and modulation of multiple signaling pathways. Although epigenetics alterations related to aging are not fully understood, this review highlights various studies towards understanding these mechanisms and their association with aging.

## Figures and Tables

**Table 1:** List of different classes of polyphenols, primary sources, their content based on weight and serving and their respective molecular functions.

Polyphenols	Polyphenol Source	Polyphenol Content Based on Weight or Volume (mg/kg wt or mg/L)	Molecular Functions	References
Phenolic acids/Hydroxybenzoic acids				
Gallic acid	Berries, pineapples, bananas, lemons and wines	40–130	Anti-oxidative, pro-oxidative, anti-inflammatory, antibacterial, antiviral, anti-melanogenic, anti-invasive and anti-proliferative	[47,48,49,50,51]
Ellagic acid	Berries, pomegranate, walnuts and pecans		Anti-oxidative, anti-inflammatory, anti-angiogenic, antimetastatic, anti-proliferative and anti-invasive	[47,52,53]
Phenolic acids/Hydroxycinnamic acids				
Caffeic acid	Kiwifruit	600–1000	Anti-diabetic, anti-carcinogenic, protective effects against UVB-induced skin damage, interleukin-10 and activation of mitogen-activated protein kinase (MAPK)	[54,55,56]
Rosmarinic acid	Herbs		Anti-oxidative, reduction of HCA formation and modulation of epigenetic changes	[47,53,57]
Ferulic acid	Aubergine	600–660	Anti-oxidative, anti-inflammatory, antibacterial, antimicrobial, antiallgergic, hepatoprotective and antiviral	[58]
Chlorogenic acid	Cherry	180–1150	Anti-oxidative, antimicrobial, anti-inflammatory, antibacterial, analgesic and antipyretic	[59,60]
Flavonoids				
Myricetin	Broccoli	40–100	Anti-oxidative, anti-inflammatory, anti-allergic properties, analgesic property, hepatoprotective and hypouricemic activities, anti-diabetic and anti-obesity properties	[61,62,63,64,65]
EGCG	Tea, apples, grapes, berries, red wine and chocolate		Anti-oxidative, anti-proliferative, suppression of growth and invasion, antiangiogenic, anti-inflammatory, inhibition of telomerase activity and lipid peroxidation and modulation of estrogen activity	[66,67,68,69,70,71]
Apigenin	Grapefruit, parsley, onion, orange, tea and wheat	20–140	Anti-oxidative, anti-mutagenic, anti-inflammatory, anti-viral, inhibition of tumor growth, anti-invasive, and anti-proliferative	[48,72,73,74]
Quercetin	Onions, broccoli, apples, apricots, berries, nuts, seeds, tea, wine and cocoa		Anti-oxidative, tumor inhibition, anti-proliferative, antimetastatic, anti-angiogenic and inhibition of lipid peroxidation	[67,70,71,72,75,76,77]
Genistein	Miso	250–900	Anti-oxidative, anti-invasive, anti-inflammatory, anti-metastatic, delay/repression of tumor development/growth and anti-proliferative	[67,70,71,72]
Stilbenes				
RES	Red wine, grapes, berries and peanuts		Anti-oxidative, anti-inflammatory, anti-proliferative and anti-estrogenic	[75,77,78,79,80,81,82]
PTER			Anti-oxidative, anti-inflammatory, anti-proliferative and modulation of lipid metabolism	[75,77,78,80,81,82]

EGCG—Epigallocatechin gallate, RES—Resveratrol, PTER—Pterostilbene.

**Table 2:** List of proteins that play a vital role in DNA methylation with their molecular activity and biological function.

DNA Methylation Enzymes	Molecular Activity	Biological Function	References
DNMT1	Replication of methylation patterns in the new strand after DNA replication Crucial for genomic imprinting Maintenance of DNA methylation during mitosis	Embryonic development Heterochromatin formation Gene silencing X chromosome inactivation Protein binding RNA bindingMethyl-CpG binding	[94,95]
DNMT3a and DNMT3b	Catalyze cytidine methylation at 5-Carbon Maintenance of DNA methylation	Crucial for *de novo* methylation in the genome Gene silencing Heterochromatin formation	[5,96,97,98]

DNMT1—DNA methyltransferase 1, DNMT3a—DNA methyltransferase 3 alpha, DNMT3b—DNA methyltransferase 3 beta.

**Table 3:** List of proteins that play a vital role in histone acetylation and methylation with their molecular activity and biological functions.

Histone Modifications	Molecular Activity	Biological Function	References
HDACs	Zinc-dependent amidohydrolases Primarily responsible for cleavage of acetyl groups from acetyl-lysine residues	DNA replication DNA repair Heterochromatin silencing Gene transcription	[104,105,106]
HATs	Utilizes acetyl-CoA for acetylation reaction	Transcriptional activation DNA repair Gene expression profiling leading to disease progression	[107]
HMTs	Methylation of lysine residues Regulation and hydrophobicity of histone tails	Gene transcription activation Gene transcription suppression DNA repair Heterochromatin formation	[108]

HDACs—Histone deacetylases, HATs—Histone acetyltransferases, HMTs—Histone methyltransferases.

**Table 4:** List of ncRNAs, their related molecular activity, and biological functions contributing to aging-related diseases.

Non-Coding RNAs	Molecular Activity	Biological Function	References
siRNAs	Controls pre-messenger RNA and regulate levels of Positive transcription elongation factor (P-TEFB) Regulates RNA Polymerase II (RNAP-II) transcription in the nucleus	Ribosomal synthesis Alternative splicing OS	[109]
miRNAs	Degradation of mRNA Genomic stability Chromatin modification left	Cell cycle regulation Cell proliferation Tumor suppression Apoptosis	[110,111,112]
lncRNAs	Expressed in intergenic regions or the promoter regions of mRNAs. Facilitates ubiquitination	Genome localization Regulates gene expression Recruitment of chromatin-modification factors Nuclear compartmentalization	[113]

siRNAs—small interfering RNAs, miRNAs—microRNAs, lncRNAs—long non-coding RNAs.

**Table 5:** Polyphenols and their specific aging-related activities across different organisms.^.^

Polyphenols	Epigenetic and Aging-Related Activity	Aging Pattern	Target Genes/Proteins	Species	References
Apigenin	Antioxidant activity Behavioral impairment ↓ Organic index Histopathological changes	Cellular senescence Organismal	Nrf2, HO-1 and NQO1, MMP-1, ↑ *p53,* ↑ *p21*,↓ Cyclins D1 and ↓ Cyclins E	*Mus musculus* *Homo sapiens*	[122,123,124]
	Repair of skin dryness ↑ Moisture content ↑ Dermal density ↑ Skin elasticity				
	anti-apoptotic and pro-apoptotic proteins imbalance ↑ Mitochondrial superoxide levels				
CUR	↑ OS DNA repair mechanisms Hormesis	Cellular senescence Organismal	*SOD1*, *SOD2* and *RAD52*, *TERT,* rADSCs and SA-β-gal	*Saccharomyces cerevisiae Rattus rattus*	[125,147,148]
	Muscle mass function ↑ Anti-oxidative properties				
	↑ Anti-oxidative properties				
EGCG	↑ Apoptosis ↓ Cellular proliferation ↓ DNMT activity ↓ HDAC activity Telomerase inhibition	Organismal	GAS1, TIMP4, ICAM1, WISP2 and hTERT	*Homo sapiens*	[130,131,132]
GES	↑ DNA repair ↓ UV-radiation exposure ↓Tyrosine kinase activity	Cellular senescence Organismal	COX-2, hTERT, p66Shc, ↑ *p16*, ↓ *p21*, SIRT1, LKB1, AMPK, ↓ *p62*, ↓ p-m-TOR and p-P70S6K	*Homo sapiens* *Mus musculus*	[135,136,137,138]
	↓ Hypermethylation ↓ DNMT1 activity ↓ DNMT3a activity				
	↑Autophagic flux				
PTER	↓ OS ↓ Mitochondrial morphological disorder	Organismal	↑ AMPK, ↑ SIRT1 and PGC-1α, ↑ REST, ↑ PSD-95 and ↑ mitochondrial porin-1	*Homo sapiens* *Mus musculus*	[140,149]
	↑ Declarative memory ↑ Working memory				
RES	↑ Myocardial performance index ↑ HDAC activity	Organismal	↓ *IGF-I,* ↑ AMPK and ↑ PGC-1α	*Mus musculus*	[142,143,150]
	↓ Inflammatory cytokines ↓ Cognitive defects Reversal of aging-associated learning and cognitive impairment				
	↓ Inflammatory cytokines ↓ Cognitive defects Reversal of aging-associated learning and cognitive impairment				
Quercetin	↑ Apoptosis ↑ OS ↓ H3K9me3 activity Delaying down-regulation of *SIRT* activity	Organismal	↓ MPF activity and ↑ PaMTH1	*Mus musculus* *Podospora anserina*	[145,146]

↓—decreased, ↑—increased, CUR—Curcumin, EGCG—Epigallocatechin-3-gallate, GES—Genistein, PTER—Pterostilbene and RES—Resveratrol.

## Abbreviations

AD	Alzheimer’s Disease
ADP	Adenosine Di Phosphate
AgeCpGs	Age-associated CpGs
AMPK	AMP-activated protein kinases
ATP	Adenosine Tri Phosphate
BUT	Sodium Butyrate
CAT	Catalase
CI	Confidence interval
CUR	Curcumin
COX-2	Cyclooxygenase-2
DADS	Diallyl disulfide
DDP	Cisplatin
DNA	Deoxyribonucleic acid
DNMTs	DNA methyltransferases
DNMT1	DNA methyltransferase 1
DNMT3a	DNA methyltransferase 3 alpha
DNMT3b	DNA methyltransferase 3 beta
DNAm	DNA methylation
DOX	Doxorubicin
EGCG	Epigallocatechin-3-gallate
EGFR	Epidermal growth factors
ER	Endoplasmic reticulum
Gadd45A	Growth arrest and DNA damage inducible alpha
GAS1	Growth arrest specific 1
GES	Genistein
GAG	Glycosaminoglycan
GPx	Glutathione peroxidase
GTPs	Green tea polyphenols
HAT	Histone acetyltransferase
HDAC	Histone deacetylase
HMT	Histone methyltransferase
HDM	Histone demethylase
HIF-1α	Hypoxia inducible factor-1α
HP1	Heterochromatin protein 1
HDFs	Human dermal fibroblasts
HUVECs	Human umbilical vein endothelial cells
H3-K27	Histone H3 on lysine 27
H3-K9	Histone H3 on lysine 9
H4-K16	Histone H4 on lysine 16
H4-K36	Histone H4 on lysine 36
ICAM1	Intracellular adhesion molecule 1
IFNγ	Interferon gamma
LCs	Langerhans cells
lncRNAs	Long non-coding RNAs
miRNAs	microRNAs
MBDs	Methyl-CpG binding domains
MPF	Maturation promoting factor
NPC	Neural progenitor cell
ncRNAs	Non-coding RNAs
OS	Oxidative stress
PGC-1α	Proliferator-activated receptor-gamma coactivator 1 alpha
PD	Parkinson’s disease
PTER	Pterostilbene
rDNA	Ribosomal DNA
RAR β	Retinoic acid receptor beta
RASSF1A	Ras-associated domain family protein 1A
RES	Resveratrol
ROS	Reactive oxygen species
SAβ-gal	Senescence-associated beta-galactosidase
Sir	Silent information regulator
siRNAs	Small interfering RNAs
SOD	Superoxide dismutase
TERT	Telomerase reverse transcriptase
TNBC	Triple negative breast cancer
TIMP 4	TIMP metallopeptidase inhibitor 1
T2D	Type 2 Diabetes
BrdU	5-Bromo-2′-deoxyuridine
5mC	5-methylcytosine
